# Perspectives on Ebola

**DOI:** 10.4269/ajtmh.14-0831

**Published:** 2015-02-04

**Authors:** Philip J. Rosenthal, Daniel G. Bausch

**Affiliations:** Department of Medicine, University of California, San Francisco, California; Department of Tropical Medicine, Tulane University School of Public Health and Tropical Medicine, New Orleans, Louisiana; U.S. Naval Medical Research Unit No. 6, Lima, Peru

An unprecedented epidemic of Ebola virus disease (EVD) unfolded in West Africa in 2014. The epidemic has been well described in the popular press and in regular reports from public health authorities. The medical literature has necessarily been slower in describing the epidemic, but comprehensive reports are now appearing, offering valuable accounts of the clinical features, epidemiology, and public health consequences of this terrifying disease. The American Society of Tropical Medicine and Hygiene (ASTMH) has been deeply involved with the EVD outbreak. Numerous ASTMH members have played major roles in addressing the epidemic, including clinicians and epidemiologists working at the front lines of the epidemic at great personal risk, public health authorities guiding control efforts in Africa and elsewhere, and drug and vaccine experts working to rush effective products to the field. The annual meeting of the ASTMH served as a forum for timely expert discussions on EVD, but also highlighted the political challenges of this particular crisis, as some experts were prevented from attending the ASTMH meeting as a result of ill-founded concerns about the consequences of their recent travel to West Africa. In this issue of the *American Journal of Tropical Medicine and Hygiene* (*AJTMH*) we offer a series of Perspectives from individuals active in addressing the EVD epidemic.

As with other large disasters, the full toll of the EVD epidemic is difficult to fathom. The numbers are clear. As of the end of 2014, nearly 20,000 cases of EVD and 7,000 deaths have been reported to the World Health Organization (WHO). These numbers are likely underestimates caused by underreporting. Furthermore, although these numbers are much lower than those seen for our greatest tropical medicine challenges, the impact of the epidemic can easily be underappreciated. EVD is quite unique, even among severe infectious diseases, in causing massive disruption to societies, and in particular to the healthcare infrastructure. In affected areas of Africa, in addition to the huge direct toll of EVD, all aspects of healthcare have been torn apart. Management and control of the most important serious infectious diseases, including neonatal infections, human immunodeficiency virus (HIV) infection, tuberculosis, malaria, and other neglected diseases have been greatly disrupted. “Band-aid” solutions, such as widespread distribution of artemisinin-based combination therapies to decrease the incidence of non-Ebola febrile illnesses, have unknown efficacy, and may cause new problems, such as selection of drug resistance and loss of community confidence in the healthcare system. Outside of Africa, responses to the EVD epidemic have often been driven by fear, misguided estimates of risk, and political considerations.

Most often, we in the scientific community appropriately focus on the data—the numbers of cases, the epidemiologic characteristics, and the efficacies of new interventions. In this process we may lose sight of the fact that a crisis such as the EVD epidemic is inherently personal. People are getting infected, suffering, and dying. In the case of this epidemic, much more so than in most humanitarian disasters, many of the victims are the healthcare workers and scientists who have willingly put themselves in harm's way to help alleviate the suffering of others. In this issue of the *AJTMH* we offer Perspectives focusing on the personal side of the epidemic, considering in particular the points of view of health workers as caregivers at risk, as patients, and as those working to improve our ability to manage and control this epidemic. Two perspectives, from Adaora Igonoh and Will Pooley, offer accounts from those who put themselves at personal risk caring for patients with EVD, and then contracted the disease themselves. Another, from Lewis Rubinson, offers an account of a potential Ebola virus exposure that led to complex consequences. Susan McClellan offers an account from one of the many non-African healthcare providers who eagerly put themselves at risk. Perspectives addressing an improved response to EVD include a discussion of how, despite some steps in the right direction, the public health community failed to best prepare for a potential hemorrhagic fever outbreak by Daniel Bausch, a consideration of rethinking discharge policy in seriously stressed EVD clinics by Tim O'Dempsey and others, and a comprehensive commentary on clinical preparedness for those providing EVD care from David Brett-Major and many others. Considering the political consequences of responses to the epidemic outside Africa, perspectives from groups led by Ramin Asgary and Piero Olliaro detail the consequences of the misguided effort of the State of Louisiana to protect public health by preventing attendance at the annual meeting of the ASTMH in New Orleans by anyone who had recently traveled to affected countries in West Africa.

The West African EVD epidemic is still unfolding. This enormous disaster is likely to have long-range consequences, with impacts on efforts to control all tropical diseases in addition to specific effects on viral hemorrhagic fever preparedness and far-reaching impacts on the affected countries. Regardless of the future overall course, the epidemic will remain deeply personal, with obvious consequences on affected patients and families, but also on health workers. We hope that the Perspectives in this issue of the *AJTMH* will help readers to appreciate the personal side of this epidemic, both as a major humanitarian disaster and as a formidable challenge for the international public health community.

